# Neurotoxins from Marine Dinoflagellates: A Brief Review

**DOI:** 10.3390/md20080016

**Published:** 2008-06-11

**Authors:** Da-Zhi Wang

**Affiliations:** State Key Lab of Marine Environmental Science/Environmental Science Research Center, Xiamen University, Xiamen 361005, P.R. China, Tel.: +86-592-2186016; Fax: +86-592-2180655; E-mail: dzwang@xmu.edu.cn

**Keywords:** Dinoflagellates, neurotoxins, voltage-gated ion channels, molecular action mechanism, paralytic shellfish poisoning, neurotoxic shellfish poisoning, ciguatera fish poisoning, azaspiracid poisoning, yessotoxin, palytoxin

## Abstract

Dinoflagellates are not only important marine primary producers and grazers, but also the major causative agents of harmful algal blooms. It has been reported that many dinoflagellate species can produce various natural toxins. These toxins can be extremely toxic and many of them are effective at far lower dosages than conventional chemical agents. Consumption of seafood contaminated by algal toxins results in various seafood poisoning syndromes: paralytic shellfish poisoning (PSP), neurotoxic shellfish poisoning (NSP), amnesic shellfish poisoning (ASP), diarrheic shellfish poisoning (DSP), ciguatera fish poisoning (CFP) and azaspiracid shellfish poisoning (ASP). Most of these poisonings are caused by neurotoxins which present themselves with highly specific effects on the nervous system of animals, including humans, by interfering with nerve impulse transmission. Neurotoxins are a varied group of compounds, both chemically and pharmacologically. They vary in both chemical structure and mechanism of action, and produce very distinct biological effects, which provides a potential application of these toxins in pharmacology and toxicology. This review summarizes the origin, structure and clinical symptoms of PSP, NSP, CFP, AZP, yessotoxin and palytoxin produced by marine dinoflagellates, as well as their molecular mechanisms of action on voltage-gated ion channels.

## 1. Introduction

Over the past few decades, the occurrence of harmful algal blooms (HABs) has increased both in frequency and in geographic distribution in many regions of the world. This has resulted in adverse impacts on public health and the economy, and has become a global concern [1–3]. It is known that certain HAB species can produce potent toxins that impact human health through the consumption of contaminated shellfish, coral reef fish and finfish, or through water or aerosol exposure [4]. In many cases, toxic species are normally present in low concentrations with no environmental or human health impacts. However, when they are present at high cell density and are ingested by filter-feeding shellfish, zooplankton, and herbivorous fishes, toxins are accumulated in these organisms and transferred to higher trophic levels through the food chain, which results in various adverse effects. It is reported that algal toxins result in more than 50,000–500,000 intoxication incidents per year, with an overall mortality rate of 1.5% on a global basis [5]. In addition to their adverse effects on human health, algal toxins are responsible for the death of fish and shellfish and have caused episodic mortalities of marine mammals, birds, and other animals depending on the marine food web [6–9].

**Figure 1:**
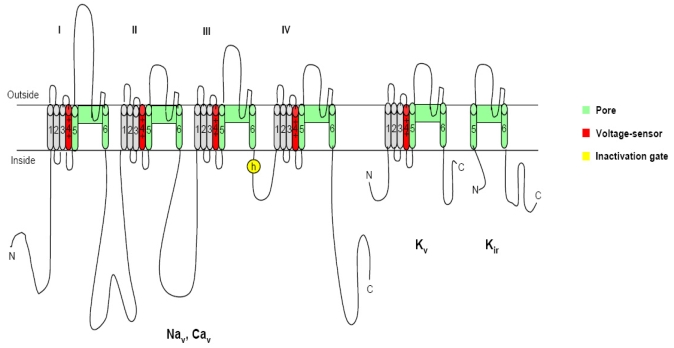
The voltage-gated channels: The different members of the ion channel family structurally related to the voltage-gated ion channels are illustrated as transmembrane folding diagrams in which cylinders represent probable transmembrane alpha helices. Green, S5–S6 pore forming segments; red, S4 voltage sensor; and gray, S1–S3 tansemembrane segments [13].

Of those causative organisms, dinoflagellates, a very large and diverse group of eukaryotic algae in the marine ecosystem, are the major group producing toxins that impact humans [4, 10]. Dinoflagellate toxins are structurally and functionally diverse, and many present unique biological activities. In the past few decades, extensive studies have been devoted to the toxicology and pharmacology of dinoflagellate toxins [11], and five major seafood poisoning syndromes caused by toxins have been identified from the dinoflagellates (Table 1): paralytic shellfish poisoning (PSP), neurotoxic shellfish poisoning (NSP), amnesic shellfish poisoning (ASP), diarrheic shellfish poisoning (DSP) and ciguatera fish poisoning (CFP). Besides these well-known poisonings, several new poisoning syndromes resulting from newly appearing dinoflagellate toxins, such as azaspiracid toxins, yessotoxin and palytoxin have been reported and characterized recently (Table 1), and this has increased global public concerns regarding dinoflagellate associated toxins. Dinoflagellate toxins can be functionally categorized as neurotoxins and hepatotoxins, according to their clinical symptoms. The neurotoxicity of dinoflagellate toxins is mediated by diverse, highly specific interactions with ion channels involved in neurotransmission (Figure 1). This paper provides a brief overview of the origin, structure and clinical symptoms of PSP, NSP, CFP, AZP, yessotoxin and palytoxin produced by dinoflagellates as well as their molecular mechanisms of action on voltage-gated ion channels.

## 2. Voltage-gated ion channels and neurotoxins

It is known that most dinoflagellate toxins are neurotoxins, which interact with the specific receptors associated with neurotransmitter receptors, or voltage-sensitive ion channels (Figure 1), resulting in the observed neurotoxicity [12]. In organisms including humans, voltage-gated ion channels, such as sodium, calcium, and potassium channels, are electrical signal generators which control contraction of muscle, secretion of hormones, sensing of the environment, processing of information in the brain, and output from the brain to peripheral tissues [13]. These channels share a common structural motif containing six transmembrane segments (S1–S6) and a pore loop (Figure 1). The voltage sensor domain consists of the S1–S4 segments with positively charged residues in the S4 segment as gating charges, while the pore is composed of the S5/S6 segments and the pore loop between them, which are gated by bending of the S6 segment at a hinge glycine or praline residue. In all of these contexts, electrical signals are conducted by members of the ion channel protein super family, a set of more than 140 structurally related pore-forming proteins [14]. Pharmacological studies have disclosed that the functions of the voltage-gated ion channel proteins can be classified into three complementary aspects: ion conductance, pore gating and regulation. These channel proteins are the molecular targets for a broad range of potent neurotoxins, which strongly alter channel functions by binding to specific receptor sites. At present, six different neurotoxin receptor sites on the channel protein have been identified on voltage-gated ion channels using various neurotoxins [15–19]. Hydrophilic low molecular mass toxins and large polypeptide toxins block the channel pore physically and prevent ion conductance. Alkaloid toxins and related lipid soluble toxins alter voltage-dependent gating through binding to intramembranous receptor sites. On the contrary, poplypeptide toxins alter channel gating through binding to extracellular sites [20, 13]. In the section below we describe the molecular mechanisms of action of seven different neurotoxins produced by dinoflagellates.

## 3. Paralytic shellfish poisoning (PSP)

**Figure 2:**
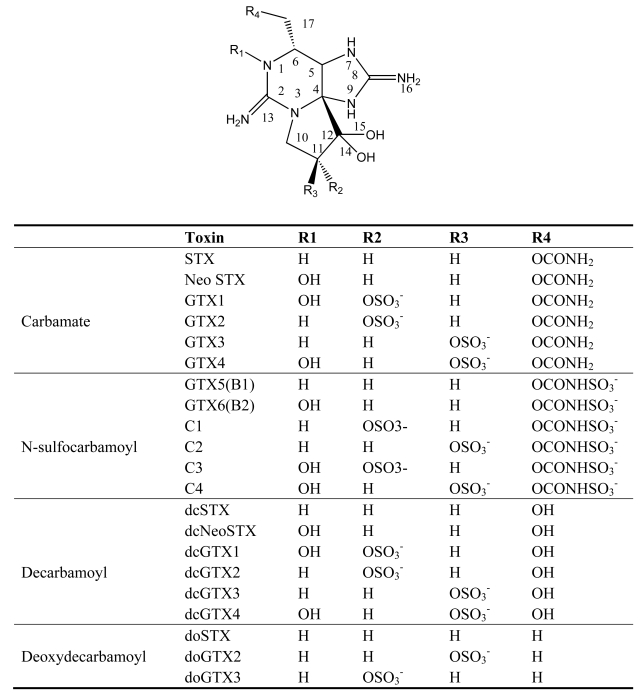
Structure and species of paralytic shellfish poisoning toxins from marine dinoflagellates [4].

PSP is a worldwide marine toxin disease with both neurologic and gastrointestinal symptoms, which is caused by the consumption of shellfish contaminated by toxic dinoflagellates [21]. The first PSP event was reported in 1927 near San Francisco, USA, and was caused by a dinoflagellate, *A. catenella*, which resulted in 102 people being ill and six deaths [22]. Since then, members of three dinoflagellate genera have been reported to be the major sources of PSP toxins: *Alexandrium, Gymnodinium,* and *Pyrodinium* [23]. Paralytic shellfish toxins (PSTs) are produced in varying proportions by different dinoflagellate species and even by different isolates within a species. PSP toxins are heat-stable and water-soluble nonproteinaceous toxins. The basic structures of PSP toxins are 3,4-propinoperhydropurine tricyclic systems. Saxitoxin and its analogues can be divided into three categories: the carbamate compounds, which include saxitoxin, neosaxitoxin and gonyautoxins 1–4; the *N*-sulfocarbamoyl compounds, which include the C and B toxins; and finally the decarbamoyl compounds with respect to the presence or absence of 1-*N*-hydroxyl, 11-hydroxysulfate, and 21-*N*-sulfocarbamoyl substitutions as well as epimerization at the C-11 position (Figure 2). In the past few decades at least 24 structurally related imidazoline guanidinium PSP derivatives have been identified and characterized from dinoflagellate species [21, 24].

Saxitoxin is the most toxic and also the most well studied among the PSP associated toxins. In mice, its LD_50_ peritoneal is 3–10 μg/kg body weight and orally is 263 μg/kg body weight. The lethal oral dose in humans is 1 to 4 mg (5,000 to 20,000 mouse units), depending on the gender and physiological condition of the patient. It is rapidly absorbed through the gastrointestinal tract and excreted in the urine. The symptoms of PSP include a tickling sensation of the lips, mouth and tongue, numbness of the extremities, gastrointestinal problems, difficulty in breathing, and a sense of dissociation followed by complete paralysis [25]. In the case of serious intoxication, PSP leads to a variety of neurological symptoms culminating in respiratory arrest and cardiovascular shock or death [26]. Saxitoxin and its analogues are very dangerous compounds, with possible military potential and have been listed by the Organization for the Prohibition of Chemical Weapons (OPCW) as a Schedule 1 chemical intoxicant, the manufacture, use, transfer and reuse of which are now strictly regulated by the OPCW (Chemical Weapons Convention, September 1998, The Hague, Netherlands).

PSP toxins are the most well known potent neurotoxins that specifically and selectively bind the sodium channels on excitable cells [27]. In 1975, Hill postulated a plugging model for the binding of the sodium channel with saxitoxin [28]. In this model, the toxin molecular penetrates rather deeply inside the channel and plugs it, having formed an ion pair with an anionic site thought to be located near the bottom of the channel. However, this model could not explain the lack of anticipated steric interactions with other structurally unfolded toxins, the gonyautoxins. Later, Kao and Walker proposed a model which placed the toxin molecules on the outside edge of the channel with the guanidinium group on the top of the channel entrance [29]. Meanwhile Shimizu also suggested a three-point binding model involving two hydrogen bonds with the ketal OHs, and ion pairing of the guanidinium group with an anionic site on the outside surface of the membrane [30]. With the success of cloning of the sodium channel [31], more precise information regarding the toxin-binding mode has arisen from the molecular biological studies of the sodium channel. PSP toxins are now regarded as blocking agents that reduce the number of conducting Na^+^ channels by occupying some site near the outer opening in a 1:1 high affinity specific receptor binding. The extracellular loop sections of S1–S2 (P-loops) of the Na^+^ channel are considered to be the PSP toxins-binding site. They bind to the site on the voltage-dependent sodium channel with high affinity (Kd~2nM), which inhibits the temporary permeability of Na^+^ ions by binding tightly to receptor site 1 on the outside surface of the membrane very close to the external orifice of the sodium channel, preventing sodium ions from passing through the membranes of the nerve cells, and thus interfering with the transmission of signals along the nerves. The resulting widespread blockade prevents impulse-generation in peripheral nerves and skeletal muscles. Saxitoxin also affects skeletal muscle directly by blocking the muscle action potential without depolarizing cells, which abolishes peripheral nerve conduction but with no curare-like action at the neuromuscular junction. Surprisingly, selective pressure from the presence of STX in the natural environment can select for mutations in the ion selectivity filter that cause resistance to these toxins in the softshell clam *Mya arenaria* [32].

## 4. Neurotoxic Shellfish Poisoning (NSP)

**Figure 3:**
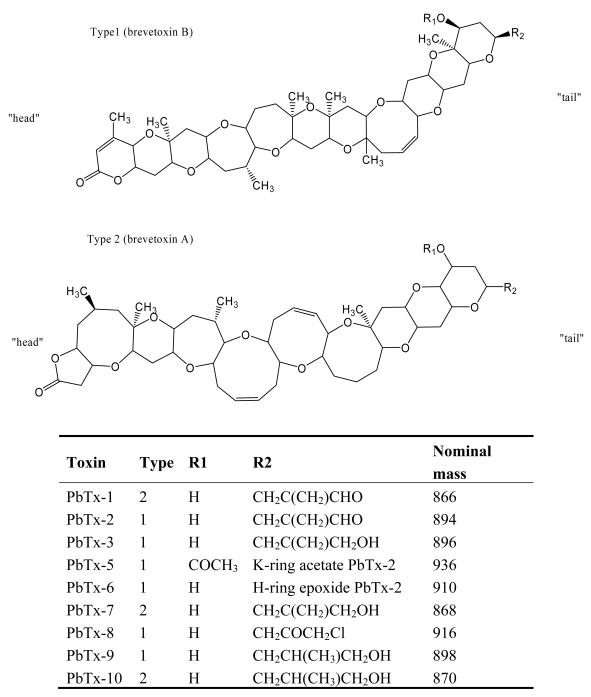
Structure and species of neurotoxic shellfish poisoning toxins from marine dinoflagellates [4].

NSP is caused by the ingestion of shellfish exposed to blooms of the dinoflagellate *Kerenia brevis* (formerly *Gymnodinium breve*) [33, 34]. This dinoflagellate species produces two types of lipid soluble toxins: hemolytic and neurotoxic [35], causing massive fish kills, bird deaths, and marine mammal mortalities [36, 37]. The neurotoxic toxins are known as brevetoxins, which are a suite of ladder-like polycyclic ether toxins. Brevetoxin congeners are of two types based on backbone structure: brevetoxin B backbone (type 1; PbTx-2, 3, 5, 6, 8, 9) and brevetoxin A backbone (type 2; PbTx-1, 7, 10) (Figure 3). Among them, PbTx-2T is the major brevetoxin produced by *K. brevis* [38]. Massive fishes are killed due to neurotoxin exposure, with the possible contribution of the hemolytic fraction. Recently neurotoxins were also found in other fish-killing flagellate species, *Chatonella marina*, *C. antiqua*, *Fibrocapsa japonica*, and *Heterosigma akashiwo* [39–41].

As with many of the known marine toxins, the brevetoxins are tasteless, odorless, and heat and acid stable (they survive heat up to 300°C). The mouse LD_50_ is 170 μg/kg body weight (0.15–0.27) intraperitoneally, 94 μg/kg body weight intravenously and 520 μg/kg body weight orally [42]. Pathogenic dose for humans is in the order of 42-72 mouse units. NSP presents itself as a milder gastroenteritis with neurologic symptoms compared with PSP. The symptoms of NSP include nausea, tingling and numbness of the perioral area, loss of motor control, and severe muscular pain [43, 44].

The mechanism of action of brevetoxins has been extensively studied, and brevetoxins are regarded as depolarizing substances that open voltage gated sodium ion channels in cell walls, leading to uncontrolled Na^+^ influx into the cell [45]. Experiments utilizing neuroblastoma cells and rat synaptosomes have shown that brevetoxins act on neurotoxin binding site 5 on the α-subunit of the voltage-dependent sodium channel in a 1:1 stoichiometry [46]. This action differs from that of PSP toxins which block the sodium channel and prevent sodium ions from passing through the membranes of nerve cells. This enhances the inward flow of Na^+^ ions into the cell by altering the membrane properties of excitable cell types, resulting in inappropriate opening of the channel under conditions in which it is normally closed, and it also inhibits channel inactivation [36, 45, 47-49]. The toxin appears to produce its sensory symptoms by transforming fast sodium channels into slower ones, which results in persistent activation and repetitive firing [50]. It was reported that brevetoxin could combine with a separate site on the gates of the sodium channel, causing the release of neurotransmitters from autonomic nerve endings. In particular, this can release acetylcholine, leading to smooth tracheal contraction, as well as massive mast cell degranulation [51]. Recently, LePage *et al.* demonstrated that brevetoxins also triggered Ca influx in rat cerebellar granule neurons. Derivatives PbTx-1, PbTx-2 and PbTx-3 produced a rapid and concentration-dependent increase in cytosolic [Ca^2+^], indicating that brevetoxin analogues display a range of efficacies to neurotoxin site 2 ligands and are activators of neurotoxin site 5, with PbTx-1 being a full agonist and other derivatives acting as partial agonists [52]. An early investigation also reported that conformational variation of brevetoxins induces a significant change in the gross shape of the molecule, which results in the loss of binding affinity and toxicity of the brevetoxins [46]

## 5. Ciguatera Fish Poisoning (CFP)

**Figure 4:**
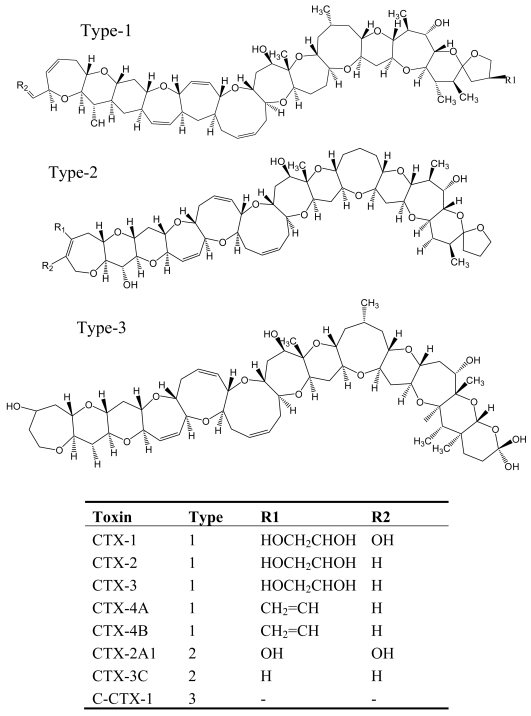
Structure and species of ciguatoxins from the dinoflagellate *G. toxicus* [62].

CFP, which is the most commonly reported marine toxin disease in the world, is caused by consumption of contaminated coral reef fishes such as barracuda, grouper, and snapper [53, 54]. It is estimated that approximately 25,000 people are affected annually by ciguatoxins and CFP is regarded as a world health problem [54]. The origin of ciguatera toxins has been identified in a dinoflagellate species, *Gambierdiscus toxicus*, which originally produces maitotoxins (MTXs), the lipophilic precursors of ciguatoxin [55]. These precursors are biotransformed to ciguatoxins by herbivorous fishes and invertebrates grazing on *G. toxicus* and then accumulated in higher trophic levels [56]. The ciguatoxins are a family of heat-stable, lipid-soluble, highly oxygenated, cyclic polyether molecules with a structural framework reminiscent of the brevetoxins [57–60], and more than 20 toxins may be involved in CFP (Figure 4) [53].

The biological activities of ciguatoxins have been studied extensively and they are regarded as the most potent activators of sodium and/or calcium fluxes in the cytoplasm in various cells. They produce more than 175 ciguateric symptoms, classified into four categories: gastrointestinal, neurological, cardiovascular and general symptoms [54, 61]. It should be emphasized that the symptoms of ciguatera vary in different oceans: in the Pacific Ocean neurological symptoms predominate, while in the Caribbean Sea the gastrointestinal symptoms dominate due to the difference in toxin composition. Ciguatoxin and maitotoxin are the two most common toxins associated with CFP, and they are the most lethal natural substances known. Pharmacological studies have revealed that CTXs activate the voltage-sensitive sodium channel at nM to pM concentrations [61]. In mice, ciguatoxin is lethal at 0.45 μg/kg ip, and maitotoxin at a dose of 0.15 μg/kg ip. Oral intake of as little as 0.1 μg ciguatoxin can cause illness in the human adult.

Ciguatoxins exert the same action mode as brevetoxins, which selectively target the common binding site 5 on the α-subunit of neuronal sodium channels. However, the affinity of ciguatoxins is higher than that of brevetoxins and, thus, the affinity of CTX-1 for voltage dependent sodium channels is around 30 times higher than that of brevetoxin. Ciguatoxins open sodium channels along the peripheral nerves, particularly at the nodes of Ranvier [62, 63], which results in an influx of Na^+^ ions, cell depolarization and the appearance of spontaneous action potentials in excitable cells. Consequently, the plasma membrane is unable to maintain either the internal environment of the cells or volume control due to the increased Na^+^ permeability, which results in alteration of bioenergetic mechanisms, cell and mitochondrial swelling and bleb formation on cell surfaces. With neurophysiological testing, significant slowing of sensory and motor nerve conduction velocities, and F wave latencies has been demonstrated [62, 64, 65]. This observation may be related to nodal swelling and internodal length and volume increase, all of which have been confirmed with *in vitro* CTX exposure [63, 66].

Studies on cardiovascular effects of ciguatoxins reveal that ciguatoxin affects voltage-dependent Na^+^ channels causing Na^+^ to move intracellularly, and normal cellular mechanisms begin to extrude sodium and take up calcium. Calcium is the intracellular trigger for muscle contraction. Although much of the increased calcium is buffered by the sarcoplasmic reticulum, it is likely that locally increased calcium concentrations increase the force of cardiac muscle contraction as is observed in ciguatoxin poisoning. A similar mechanism of ciguatoxin-induced intracellular transport of calcium occurs in intestinal epithelial cells. The increased concentration of intracellular calcium induced by ciguatoxin acts as a second messenger in the cell, which disrupts important ion-exchange systems, resulting in fluid secretion and symptoms of diarrhea [67].

**Figure 5:**
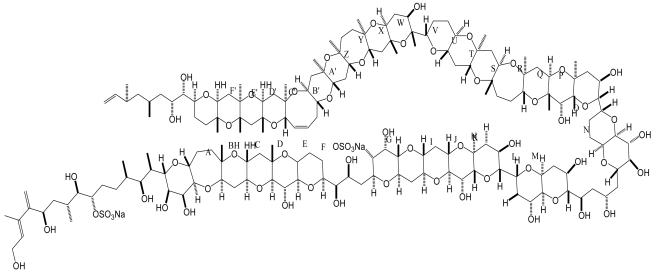
Structure of maitotoxin from the dinoflagellate *G. toxicus* [120].

Maitotoxin, another important neurotoxin involved in CFP, is a water soluble, ladder-shaped polycyclic molecule with numerous hydroxyl groups and sulfate groups (Figure 5). Three forms of MTX, MTX-1, MTX-2 and MTX-3 have been identified from *G. toxicus* [68]. MTX has been proved to be the most potent toxin identified on a weight basis: the LD_50_ of MTX in mice is less than 0.2 μg/Kg (intraperitoneally) and it is at least 5-fold more toxic than tetrodotoxin. Pharmacological studies demonstrate that MTX is a potent activator of voltage-gated calcium channels which stimulates the movement of Ca^2+^ ions across biomembranes in a wide variety of organisms. As a consequence of Ca^2+^ influx, maitotoxins can produce several effects: hormone and neurotransmitter secretion, phosphoinositides breakdown, and activation of voltage gated Ca^2+^ channels due to membrane depolarization. However, the primary target of MTX still remains undefined and the molecular mechanism of action is not clear. It is postulated that MTX might cause a shift in voltage-dependence of gating that favors opening of voltage-gated calcium channels at resting membrane potentials. However, MTX activates voltage-gated calcium channels indirectly via membrane depolarization as a consequence of activating a nonselective cation current [69]. Recently, Kakizaki *et al.* reported that maitotoxin induced a profound increase in the Ca^2+^ influx into cultured brainstem cells after a brief lag period, indicating Ca^2+^ permeability by acting on the calcium channel in an open state and preventing its closing [70].

## 6. Azaspiracid Shellfish Poisoning (AZP)

**Figure 6:**
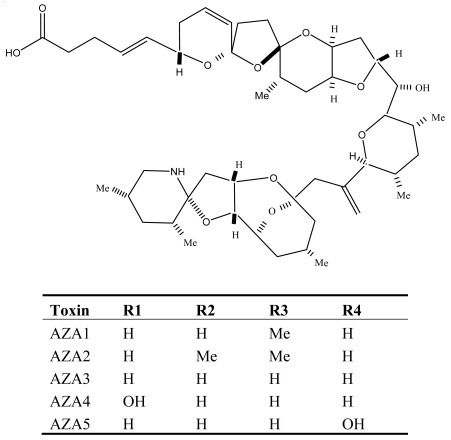
Structure and species of azaspiracid poisoning toxins from marine dinoflagellates [121].

Azaspiracid poisoning (AZP), first reported from the Netherlands but later becoming a continuing problem in Europe [71], is a newly identified marine toxin disease. It is caused by consumption of contaminated shellfish associated with the dinoflagellate *Protoperidinium crassipes*, which can produce high intracellular concentrations of azaspiracid (AZA1), a lipophilic, polyether toxin. Nowadays about one dozen derivatives (AZA2 to 11) of azaspiracid (AZA1) have been identified and characterized from *P. crassipes* and contaminated shellfish [72–74]. AZAs differ significantly from other dinoflagellate toxins, in that they have unique structural features characterized by a tri-spiro assembly, an azazpiro ring fused with a 2,9-dinoxabicyclo[3.3.1] nonane and a terminal carboxylic acid group (Figure 6).

The symptoms of AZP include nausea, vomiting, severe diarrhea and stomach cramps. Neurotoxic symptoms were also observed [72, 75, 76]. However, the extremely limited availability of the pure toxins has impeded the necessary investigations of AZP. Some experiments carried out with mice showed that AZP, unlike okadaic acid (OA) and its analog, dinophysistoxin-1, which need an initiator [77], can cause lung tumor formation during repeated administration or after withdrawal of AZP without the combined use of any initiator [78]. Also the toxin can cause necrosis in the lamina propria of the small intestine and in lymphoid tissues such as the thymus, spleen and Peyer's patches [78]. The action mechanism of AZAs is unknown at present. Some studies indicate that AZAs might have different targets, since AZA1 and AZA2 increase [Ca^2+^]_i_ by activation of Ca^2+^ -release from internal stores and Ca^2+^-influx, while AZA3 induces only Ca^2+^-influx. AZA5 does not modify intracellular Ca^2+^ homeostasis. Recent investigation of the effect of AZA4 on cytosolic calcium concentration [Ca^2+^]_i_ in fresh human lymphocytes demonstrated that AZA4 inhibits store-operated Ca^2+^ channels (SOC channels) and Ca^2+^ influx and that this process is reversible [76]. It was postulated that AZA4 inhibits SOC channels by direct interaction with the channel pore, with another region of channel protein or with a closely associated regulatory protein and it was also found that AZA4 acts through another type of Ca^2+^ channel, probably some non selective cation channel usually activated by MTX [76]. AZA groups are novel inhibitors of Ca^2+^ channels, SOC and non-SOC channels. Further study is needed to determine the primary target and the molecular mechanisms of action of AZAs on Ca^2+^ channels.

## 7. Yessotoxin (YTX)

YTX and it analogues, which are disulphated polyether compounds of increasing occurrence in seafood, were originally isolated from the scallop *Patinopecten yessoensis*, collected at Mutsu Bay, Japan [79]. Since then, YTXs have been found in Europe, South America and New Zealand, and become a worldwide concern due to its potential risk to human health. YTXs were produced by three dinoflagellate species, *Protoceratium reticulatum*, *Lingulodinium polyedrum* and *Gonyaulax spinifera* [80–83].

**Figure 7:**
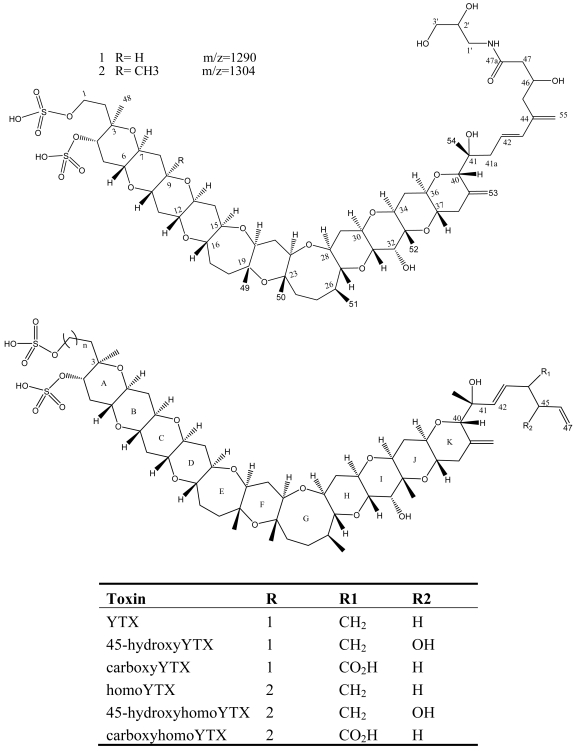
Structure and species of yessotoxins from marine dinoflagellates [122].

YTX and its derivatives, 45-hydroxy YTX (45-OH-YTX), 45,46,47-trinor YTX, homo YTX, and 45-hydroxyhomo YTX [84, 85] are disulfated polyether lipophilic toxins originally isolated from Japanese scallops (Figure 7) [80]. Recently several new YTX analogues: carboxyyessotoxin (with a COOH group on the C_44_ of YTX instead of a double bond); carboxyhomoyessotoxin (with a COOH group on the C_44_ of homoYTX instead of a double bond); 42,43,44,45,46,47,55-heptanor-41-oxo YTX and 42,43,44,45,46,47,55-heptanor-41-oxohomo YTX in Adriatic mussels (*M. galloprovincialis*) have been identified in dinoflagellates [86, 87].

Originally, YTXs were classified among the toxins responsible for DSP, mainly because they appear and are extracted together with the DSP toxins, OA and the dinophysistoxins (DTXs) [80]. However, YTXs are proved to be not diarrheogenic compared to OA and its derivatives, the DTXs, which cause intestinal fluid accumulation or inhibition of protein phosphatase 2A. Terao *et al.* demonstrated that the heart is the main target organ of YTXs in mice [88]. Toxicological studies indicated that acute oral administration at doses up to 10 mg/kg YTX or repeated (seven days) oral exposure to high (2 mg/kg/day) doses of the toxin caused no mortality nor strong signs of toxicity in mice [89–91]. YTX caused motor discoordination in the mouse before death due to cerebellar cortical alterations [90, 92, 93]. Histopathological study revealed that YTX provoked alterations in the Purkinje cells of the cerebellum, including cytological damage to the neuronal cell body and change in the neurotubule and neurofilament immunoreactivity [93].

Recently it was demonstrated that YTX is a potent neurotoxin to neuronal cells. However, the action site and the mechanism are unknown [94]. YTX was observed to induce a two-fold increase in cytosolic calcium in cerebellar neurons that was prevented by the voltage-sensitive calcium channel antagonists nifedipine and verapamil. These results suggest YTX might interact with calcium channels and/or sodium channels directly. Previous studies also showed that YTX activated nifedipine-sensitive calcium channels in human lymphocytes [95], and YTX was postulated to activate non-capacitative calcium entry and inhibit capacitive calcium entry by emptying of internal calcium stores.

## 8. Palytoxin (PTX)

PTX is a polyhydroxylated compound that shows remarkable biological activity at an extremely low concentration [96]. This toxin was first isolated from the soft coral *Palythoa toxica* and subsequently from many other organisms such as seaweeds and shellfish. Recently, palytoxin was also found in a benthic dinoflagellate, *Ostrepsis siamensis*, which caused blooms along the coast of Europe [97–102], extensive death of edible mollusks and echinoderms [99, 100] and human illnesses [98, 99]. Cases of death resulting from PTX have been reported to be due to consumption of contaminated crabs in the Philippines [103], sea urchins in Brazil [104] and fish in Japan [105–107]. PTX has become of worldwide concern due to its potential impact on animals including humans.

**Figure 8:**
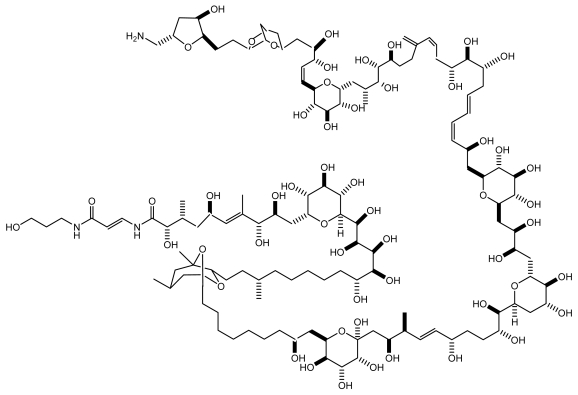
Structure of palytoxin from marine dinoflagellates [123].

PTX is a large, very complex molecule with both lipophilic and hydrophilic regions, and has the longest chain of continuous carbon atoms in any known natural product (Figure 8). Recently several analogues, ostreocin-D (42-hydroxy-3, 26-didemethyl-9,44-dideoxypalytoxin) and mascarenotoxins were identified in *O. siamensis*. PTX is regarded as one of the most potent toxins so far known [108], the LD_50_s 24 h after intravenous injection vary from 0.025 μg/kg in rabbits and about the same in dogs to 0.45 μg/kg in mice, with monkeys, rats and guinea pigs around 0.9 μg/kg. Toxic symptoms include fever inaction, ataxia, drowsiness, and weakness of limbs followed by death.

Over the past few decades much effort has been devoted to define the action mechanisms of PTXs, however these have not been identified. Pharmacological and electrophysiological studies have demonstrated that PTXs act as a haemolysin and alter the function of excitable cells. PTX selectively binds to the Na^+^, K^+^-ATPase with a Kd of 20 pM [109] and transforms the pump into a channel permeable to monovalent cations with a single-channel conductance of 10 pS [110–113]. Presently, three primary sites of action of PTXs have been postulated: PTX first opens a small conductance, non-selective cationic channel which results in membrane depolarization, K^+^ efflux and Na^+^ influx. Subsequently, the membrane depolarization may open voltage dependent Ca^2+^ channels in synaptic nerve terminals, cardiac cells and smooth muscle cells, while Na^+^ influx may load cells with Na^+^ and favor Ca^2+^ uptake by the Na^+^/Ca^2+^ exchanger in synaptic terminals, cardiac cells and vascular smooth muscle cells. Then the increase of [Ca^2+^l_i_ stimulates the release of neurotransmitters by nerve terminals, of histamine by mast cells and of vasoactive factors by vascular endothelial cells as a signal. It also induces contractions of striated and smooth muscle cells. Additional effects of a rise in [Ca^2+^]_i_ may be activation of phospholipase C [114] and phospholipase A2 [115]. There are reports that PTX opens an H^+^ conductive pathway which results in activation of the Na^+^/H^+^ exchanger [116, 117]. Other investigators suggest that PTX raises [Ca^2+^]_i_ independently of the activity of voltage dependent Ca^2+^ channels and Na^+^/Ca^’+^ exchange [118]. The last two actions might act as the opening of H^+^ specific and Ca^2+^ specific channels. Overall, PTX might posses more than one site of action in excitable cells and act as an agonist for low conductance channels conducting Na^+^/K^+^, Ca^2+^ and H^+^ ions.

## 9. Summary

This paper briefly outlines the origin, structure, symptoms and molecular action mechanisms of neurotoxins produced by marine dinoflagellates. These toxins vary in chemical structure and mechanism of action, and produce very distinct biological effects, which provides a potential application of these toxins in pharmacology and toxicology. However, some of them have not been well studied due to the limited supply of pure toxins and their molecular action mechanisms are unknown. Moreover, novel species of neurotoxins produced by dinoflagellates have been found and identified, which provide a challenge for the characterization of their toxin mechanisms and their effects on marine organisms and humans. Further work using the cell-based approach is needed to determine the precise mode of action of these novel neurotoxins from marine dinoflagellates.

## Figures and Tables

**Table 1:** Seafood poisonings caused by neurotoxins identified from marine dinoflagellate species.

Type of poisoning	Toxins	Sources of toxins	Primary vector	Action target	Ref.
PSP	Saxitoxins and gonyautoxins	*Alexandrium* spp., *Gymnodinium* spp., *Pyrodinium* spp.	Shellfish	Voltage-gated sodium channel 1	23, 27–29
NSP	Brevetoxins	*Kerenia brevis, Chatonella marina, C. antiqua, Fibrocapsa japonica, Heterosigma akashiwo*	Shellfish	Voltage-gated sodium channel 5	33, 39–41, 46, 52
	Yessotoxins	*Protoceratium reticulatum, Lingulodinium polyedrum Gonyaulax spinifera*	Shellfish	Voltage-gated calcium/sodium channel?	94–95
CFP	Ciguatoxins	*Gambierdiscus toxicus*	Coral reef fish	Voltage-gated sodium channel 5	55, 62, 63
CFP	Maitotoxins	*Gambierdiscus toxicus*	Coral reef fish	Voltage-gated calcium channel	69, 70
AZP	Azaspiracids	*Protoperidinium crassipes*	Shellfish	Voltage-gated calcium channel	72, 76
Palytoxin poisoning	Palytoxins	*Ostrepsis siamensis*	Shellfish	Na^+^-K^+^ ATPase	97, 109–111

Notes: PSP, paralytic shellfish poisoning; NSP, neurotoxic shellfish poisoning; CFP, ciguatera fish poisoning, AZP, azaspiracid poisoning.
